# FfF: Foundations for Food (security) are cellular

**DOI:** 10.1007/s00709-023-01869-z

**Published:** 2023-06-02

**Authors:** Peter Nick, Joseph Gottlieb Kölreuter

**Affiliations:** Joseph Gottlieb Kölreuter Institute for Plant Sciences, Karlsruhe Institute of Technology, Karlsruhe, Germany

Over decades, the Green Revolution has increased the global supply of staple crops at a tremendous pace. However, since around 2 decades, the production per capita has been decreasing again. This is mainly due to the impact of climate change. For instance, the progress in wheat breeding in North America has already fallen behind the yield losses caused by climate change (Zhang et al. 2022). While in the public perception, climate change is mainly understood in terms of heat and drought, its reality is far more complex. Climate change comes in many colours and may also mean stress by flooding or lodging due to untimely heavy rain, cold stress due to blurred seasonality, or low light due to skies that are overcast in consequence of human pollution. This message has meanwhile arrived in breeding programmes that no longer target on high yield alone, but progressively shift towards sustainable yield, even under stress. It is possible to perform resilience breeding without much of a conceptual understanding of the trait behind the breeding goal, just by pragmatically combining sources from resilient germplasm. However, while this is still the predominant approach, a deeper insight into the mechanisms of resilience will help to make breeding more efficient. Mechanistic understanding allows for selecting germplasm in a way that the traits are acting synergistically, allows for predicting the costs of resilience in order to minimise them, and allows for marker-assisted strategies that help to render the introgression of resilience into high-yielding varieties fast and efficient. As illustrated by two contributions to the current issue, this mechanistic insight must be based on a thorough understanding of the cellular foundations of resilience.

The contribution by Wang et al. (2023) is addressing the mitochondrial response to waterlogging stress in wheat roots. As in many agricultural systems shaped by a monsoon season, South China is depending on paddy-rice culture during the rainy season, while a second crop is grown during the drier autumn and winter seasons. As a winter crop, wheat plays a dominating role, being harvested in spring, before the next monsoon season starts. For the quantity and quality of the yield, the warm and dry weather in March and April is crucial. However, over the last few years, untimely and massive rains have caused tremendous damage by flooding. In fact, this phenomenon (which is also impacting wheat harvests in India) represents a clear case of blurred seasonality as a consequence of climatic shifts. The authors address this challenge by investigating what actually happens when wheat roots are exposed to flooding. In their previous work, they have shown that flooding will accelerate programmed cell death in the endosperm (Qi et al. 2018). This process is triggered by the formation of a so-called mitochondrial permeability transition pore, such that cytochrome can leak out into the cytoplasm. When waterlogging impairs the tightness of the inner membrane, this will trigger the autophagy of mitochondria, which will reduce the level of oxidative stress and prevent the accumulated starch from being consumed for inefficient respiration. Under prolonged stress, programmed cell death will ensue. Organelle autophagy has evolved early in eukaryotic evolution and can be interpreted as a last resort for cellular survival. Therefore, the underlying signal transduction shares certain commonalities with autophagy in fungal and mammalian cells while exhibiting plant-specific aspects as well (for review, see Reumann et al. 2010). In the current study, authors addressed the response of the challenged root itself. They observed the induction of autophagy-related genes and a progressive proteolysis of cytochrome c and COX II, followed by mitophagy. Mitophagy in response to water logging could be blocked by Cyclosporin A, an inhibitor of the transition pore, and evoked in the absence of stress by CCCP, an ionophore impairing the tightness of the inner membrane. Thus, it seems to be the permeabilisation of the inner membrane that is necessary and sufficient to induce mitophagy. It will be interesting to investigate in the future how mitophagy in the hypoxic root itself is linked with the mitophagy in the endosperm during grain filling. What are the systemic signals? And how do these phenomena help or hinder the adaptation of wheat to untimely rain?

Also, the contribution by Panda et al. (2023) addresses the effect of stress on grain filling, this time in rice, the most important staple food in Asia. The stress here is low-light stress during the grain-filling period. This can be due to a prolonged monsoon season, but it can also be caused by overcast skies in consequence of air pollution, or by planting rice in excessive density. As a result, the translocation of carbohydrates from the leaves into the panicle is impaired, leading to a lower accumulation of starch in the endosperm. In fact, they can show that key enzymes of sink carbohydrate metabolism, such as starch synthase, are repressed under low-light stress. Based on a previously reported correlation between grain filling and auxin content (Wang et al. 2006), the authors investigated a potential relationship between low-light stress and changes of auxin homeostasis. Here, they can show a significant reduction of auxin levels in the spikelet along with a significant downregulation of the auxin synthesis gene YUCCA11. They can show further that RGB1, the only trimeric G-protein known in plants and known to be essential for auxin signalling, is downmodulated as well. To further corroborate this connection, they compare two stress-susceptible genotypes of rice with two genotypes that can cope better with low-light stress. They can show that the two tolerant genotypes can sustain YUCCA11 expression, auxin accumulation, and G-protein expression more efficiently as compared to the susceptible genotypes. This genetic variation tells that it should be possible to improve resistance to low-light stress by breeding. The identification of YUCCA11 as a relevant player enables strategies for searching for favourable promoter alleles and introgressing them into high-yielding varieties using marker-assisted selection. In addition, this work identifies auxin homeostasis as relevant factor for grain filling. In the next step, it would be relevant to dissect the mechanism, by which low light perceived in the leaves leads to changed auxin homeostasis in the spikelet. A link (however, inverted to the situation here) between excessive canopy density and changes of auxin homeostasis has been reported earlier, for the so-called shade avoidance response, where plants stimulate internode length when they sense close neighbours due to altered ratios between red and far-red light. It might be rewarding to test, whether the spikelet just responds to the altered source-sink relation or whether photomorphogenetic events in the leaves play a role as well.

Both contributions are motivated by the attempt to understand the mechanisms behind the impact of climate change upon cereal productivity. Both address cellular mechanisms (mitochondrial autophagy, regulation of carbohydrate partitioning by auxin), and both come up with a working hypothesis that not only leads to interesting questions guiding future research but also defines interesting molecular candidates that can be targeted by future breeding programmes. The lesson we can learn from this work is that it is always worth to invest into a deeper mechanistic understanding of stress phenomena, not only for the sake of science itself but also for the sake of hypothesis-driven application.
